# Inflammation in Duchenne Muscular Dystrophy–Exploring the Role of Neutrophils in Muscle Damage and Regeneration

**DOI:** 10.3390/biomedicines9101366

**Published:** 2021-10-01

**Authors:** Ankita Tulangekar, Tamar E. Sztal

**Affiliations:** School of Biological Sciences, Monash University, Melbourne 3800, Australia; Ankita.Tulangekar@monash.edu

**Keywords:** Duchenne muscular dystrophy, DMD, inflammation, neutrophils, myeloperoxidase, neutrophil elastase

## Abstract

Duchenne muscular dystrophy (DMD) is a severe and progressive, X-linked, neuromuscular disorder caused by mutations in the *dystrophin* gene. In DMD, the lack of functional dystrophin protein makes the muscle membrane fragile, leaving the muscle fibers prone to damage during contraction. Muscle degeneration in DMD patients is closely associated with a prolonged inflammatory response, and while this is important to stimulate regeneration, inflammation is also thought to exacerbate muscle damage. Neutrophils are one of the first immune cells to be recruited to the damaged muscle and are the first line of defense during tissue injury or infection. Neutrophils can promote inflammation by releasing pro-inflammatory cytokines and compounds, including myeloperoxidase (MPO) and neutrophil elastase (NE), that lead to oxidative stress and are thought to have a role in prolonging inflammation in DMD. In this review, we provide an overview of the roles of the innate immune response, with particular focus on mechanisms used by neutrophils to exacerbate muscle damage and impair regeneration in DMD.

## 1. Introduction

Dystrophin is required to maintain muscle cell integrity during the repeated cycles of muscle contraction and relaxation associated with muscle activity [1]. Dystrophin functions by providing an anchor between the actin cytoskeleton and the Dystrophin-associated glycoprotein complex (DGC) at the muscle cell membrane (sarcolemma) [2]. In turn, the extracellular domain of the DGC binds to the extracellular matrix protein laminin, thereby connecting the intracellular cytoskeleton to the extracellular matrix proteins and maintaining the stability of the muscle cell [3,4].

Duchenne muscular dystrophy (DMD) is a severe and progressive X-linked disorder, affecting approximately 1 in 3600 males during early childhood. It leads to a shortened lifespan of an individual due to respiratory or cardiovascular failure caused by progressive muscle weakness [4]. DMD is caused by mutations in the *dystrophin* gene resulting in a loss of functional dystrophin protein, leaving the sarcolemma unstable, and the muscle fibers prone to damage after skeletal muscle contraction [5,6]. In DMD patients, muscle weakness results from continuous rounds of muscle damage followed by regeneration [7]. The regenerative capacity of the muscle fibers, however, is eventually exhausted, leading to impaired muscle repair. This is subsequently followed by muscle fiber necrosis and gradual replacement of muscle fibers with adipose and connective tissue leading to fibrosis, which further impedes muscle regeneration [3,8,9].

## 2. Persistent Activation of the Immune System Induces a Chronic Inflammatory State in DMD

The innate immune system, including macrophages, neutrophils, natural killer (NK) cells, and dendritic cells (DCs), serves as the body’s first line of defense [10], and is activated in response to pathogens, tissue injury or damage [11]. Often, the initiation of the inflammatory process stimulates tissue repair, whereby cell debris is removed from the inflamed sites by immune cells and tissue homeostasis can be restored.

In healthy muscles, muscle damage induced by mechanical stress initiates inflammation and recruitment of immune cells to the sites of damage. Upon activation, the recruited immune cells proliferate and secrete chemokines and cytokines that promote a local inflammatory response. This, coupled with increased oxidative stress at the site of muscle damage, attracts additional effector immune cells [5,12]. Simultaneously, the innate immune cells further assist in tissue regeneration by inducing proliferation and maturation of satellite cells, which are the precursor cells of myofibers [13,14,15].

**Figure 1:**
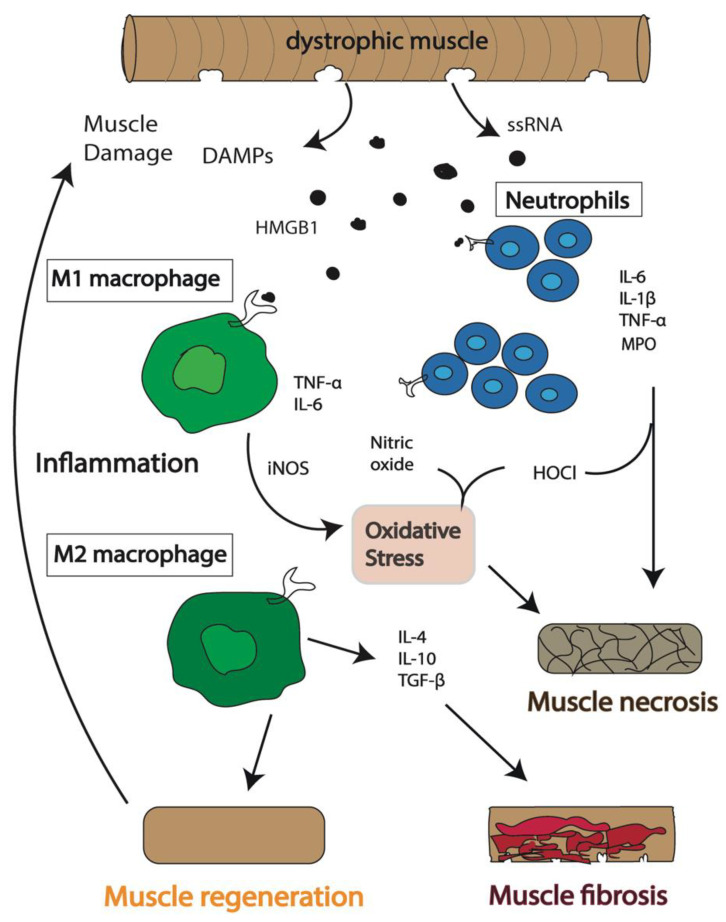
Schematic of the immunological events following muscle damage in Duchenne muscular dystrophy (DMD). An inflammatory response is activated in dystrophic muscle cells, and immune cells, including neutrophils and macrophages, are recruited to the sites of damage. The expression of inducible nitric oxide synthase (iNOS), myeloperoxidase (MPO), hypochlorous acid (HOCl), and pro-inflammatory cytokines, including interleukin (IL) 6 (IL-6), tumor necrosis factor alpha (TNF-α) and IL-1β, followed by anti-inflammatory cytokines, including IL-10, IL-4 and transforming growth factor beta (TGF-β), combined with the release of DAMPs including single stranded RNA (ssRNA) and high mobility group box protein 1 (HMGB1), initially results in regeneration of the muscle. However, continuous release of cytokines and DAMPs results in prolonged inflammation. This chronic inflammatory condition leads to impaired muscle repair followed by necrosis of muscle cells and accumulation of excessive fatty connective tissue leading to fibrosis.

In DMD, the prolonged activation of the innate immune response leads to excessive inflammation resulting in chronic inflammation, often causing additional tissue damage. The continuous contraction-induced membrane damage results in leakage of cytoplasmic contents from the muscle cells, such as creatine kinase and damage associated molecular patterns (DAMPs). These are normally sequestered intracellularly but, when released into the extracellular space, they are recognized by, and activate, the innate immune cells [16]. The continuous release of DAMPs, including high mobility group box protein 1 (HMGB1), adenosine triphosphate ATP, single-stranded RNA ssRNA, hyaluronic acid, and heat shock proteins (HSPs), in response to the ongoing cycles of damage and regeneration in dystrophic muscle, prolongs the activation and recruitment of immune cells inducing a chronic inflammatory state [7,17]. Ultimately, this leads to the formation of fatty and connective tissue permanently limiting muscle contraction [6,9,18] (Figure 1).

## 3. Which Immune Cells Are the Key Players in DMD Pathogenesis?

Recognition of DAMPs by their cognate receptors activates multiple downstream signaling pathways that exacerbate muscle damage in DMD. Many of these molecular pathways are key modulators of inflammation and oxidative stress, which are underlying pathological events in DMD [3,19]. DAMPs have been shown to influence the recruitment and function of immune cells, including macrophages and neutrophils, at the site of damage in dystrophic muscle [17]. These DAMPs are recognized by a variety of pathogen recognition receptors, or PRRs, including toll-like receptors (TLR2/4/7), which further activate downstream signaling pathways that elicit a prolonged inflammatory response in DMD [7,17]. Remarkably, preventing this interaction in *mdx* mice by deleting TLR2 or providing a TLR7/9 antagonist, significantly reduced muscle inflammation and improved skeletal muscle function, demonstrating a role of TLR-DAMP interactions in promoting muscle degeneration in DMD [20,21]. Furthermore, increased levels of HMGB1 in *mdx* mice are reported to promote inflammation and muscle degeneration, indicating the importance of identifying additional DAMPs which have the potential to act as biomarkers for DMD [22].

Many signaling pathways with essential roles in inflammation and innate immunity in healthy muscle are significantly dysregulated in DMD. The major drivers of chronic inflammation in DMD are the nuclear factor kappa B (NF-κB) pathway, together with c-Jun NH2-terminal kinase (JNK) and interferon regulatory factors (IRFs). These are activated by cytokines such as tumor necrosis factor alpha (TNF-α) and interleukin (IL) 6 (IL-6), which subsequently initiate the downstream myeloid differentiation primary response 88 (MyD88)-dependent pathway. This, in turn, activates the IκB kinases (IKKs) and the mitogen-activated kinases (MAPKs), and ultimately upregulates NF-κB and activator protein 1 (AP-1) signaling pathways [23]. These transcription factors translocate to the nucleus and induce the expression of pro-inflammatory genes, including chemokines, cytokines, cell adhesion molecules and enzymes [16,23]. Upregulation of IL-6 promotes inflammation and reduces the muscle satellite cell populations required for muscle regeneration in DMD [23,24,25]. Therefore, multiple NF-κB inhibitors including Edasalonexent (CAT-1004) and Flavocoxid have been used to reduce inflammation in DMD and are currently in Phase 2 and Phase 3 clinical trials, respectively [26,27]. Furthermore, transient administration of a STAT3 inhibitor in *mdx* mice improved the overall regenerative capacity of the muscle [28]. Additionally, treatment with the glucocorticoid, dexamethasone, resulted in reduced expression of miR-379, a miRNA involved in mitochondrial metabolism which was shown to be dysregulated in a GRMD dog model for DMD. This highlights the potential for anti-inflammatory drugs to also aid regeneration in DMD by restoring mitochondrial function in dystrophic muscle [29].

### 3.1. Macrophages

Macrophages are one of the major innate immune cells and have a number of diverse roles in muscle, ranging from defense against potentially damaging molecules, to tissue repair and regeneration [13,30]. Macrophages are a heterogenous population of immune cells with a broad spectrum of subtypes displaying distinct functions. They exhibit remarkable plasticity, and their physiology is strongly influenced by the microenvironment in which they are activated [31]. Macrophage subtypes on extreme ends of this spectrum are represented by pro-inflammatory (M1-like) and anti-inflammatory (M2-like) macrophages [3]. In DMD, macrophages are one of the most abundant cells that accumulate at the sites of muscle breakage [32]. The asynchronous and continuous cycles of muscle damage and repair occurring in DMD creates a constant presence of M1 and M2 macrophages at the sites of damage [31,33], and a self-sustaining activation of the innate immune system.

When muscle breakage occurs, pro-inflammatory M1 macrophages are required to initiate the inflammatory process that will promote repair and regeneration. M1 macrophages use PRRs to recognize the harmful endogenous molecules that are released from the cell and induce the onset of inflammation [3,31]. However, in DMD the continuous recruitment of M1 macrophages leads to a chronic inflammatory state producing high concentrations of proinflammatory cytokines such as TNF-α, IL-6, and IL-1β. These can induce the production of inducible nitric oxide synthase (iNOS) that catalyzes the production of nitric oxide, which alone or in combination with other oxidizing radicals, is known to significantly damage the dystrophic muscle [3,34]. High concentrations of these free radicals cause cell lysis and increase damage of the surrounding tissues producing chronic inflammatory conditions (Figure 1).

In contrast to the pro-inflammatory subtype, anti-inflammatory or pro-regenerative M2 macrophages release anti-inflammatory cytokines, including IL-10 and arginase which reduce iNOS production (stimulated by M1 macrophage activation) and promote muscle repair [3,34]. M2 macrophage populations regulate skeletal muscle regeneration by increasing the proliferation and maturation of muscle progenitor cells including satellite cells and fibroblasts [13,14]. Satellite cells comprise stem cells and progenitors which have the capacity to either undergo myogenic reprogramming, generate new myogenic progenitors required for muscle repair or to self-renew upon activation. Over time, in healthy, aged muscle, satellite cell numbers decline and there is reduced entry into the cell cycle, leading to decreased quantities of both stem and progenitor cell populations and an inability to effectively contribute to muscle regeneration [15].

However, in DMD muscle, the constant requirement for muscle repair leads to the production of a defective population of muscle progenitor cells impairing muscle regeneration [35]. In fact, studies have showed that despite the number of satellite cells being elevated in *mdx* mice, the dystrophic environment promotes dysregulation of satellite cell function with many displaying impaired asymmetric cell division, an inability to establish cell polarity and reduced myogenic potential [15,36]. In these dystrophic conditions, aged muscle satellite cells have been shown to convert from a myogenic to a fibrotic lineage and are thought to be a main source of fibroblasts. Therefore, the impaired regenerative capacity of dystrophic muscle is not just due to an exhaustion of muscle stem cells but also results from a loss of proper satellite cell function which likely enhances fibrosis. This, combined with continual activation of M2 macrophages in chronic inflammatory conditions, causes the accumulation of extracellular matrix (ECM) via the continual release of the pro-fibrotic protein, transforming growth factor beta (TGF-β) [18]. Excessive connective tissue proteins, such as collagen, lead to a permanent replacement of the muscle fibers with fatty and connective tissue causing fibrosis [3,6,8] (Figure 1). The contribution of each macrophage subtype to DMD pathogenesis is still unclear; however, the balance between M1 and M2 macrophage populations remains a critical factor to reduce chronic inflammatory processes and maximize the regenerative potential of the muscle. Interestingly, inhibition of myostatin, part of the TGF-β signaling pathway, improved muscle growth in *mdx* mice. However, it had detrimental effects on the testis and significantly reduced both the quality and quantity of sperm in *mdx* mice, highlighting the importance of testing therapies for DMD for off-target effects on other non-muscle tissues [37].

### 3.2. Neutrophils

Neutrophils, also known as polymorphonuclear leukocytes, are the most abundant circulating immune cells involved in various immunological and inflammatory events [38]. Neutrophils are produced in the bone marrow from a hematopoietic stem cell pool, which undergoes transformation from immature to mature neutrophils, and are then released into the blood stream where they can be mobilized to the site of inflammation [39]. Neutrophils are responsible for clearing up the cell debris during tissue injury and defense against invading microorganisms [40].

**Figure 2:**
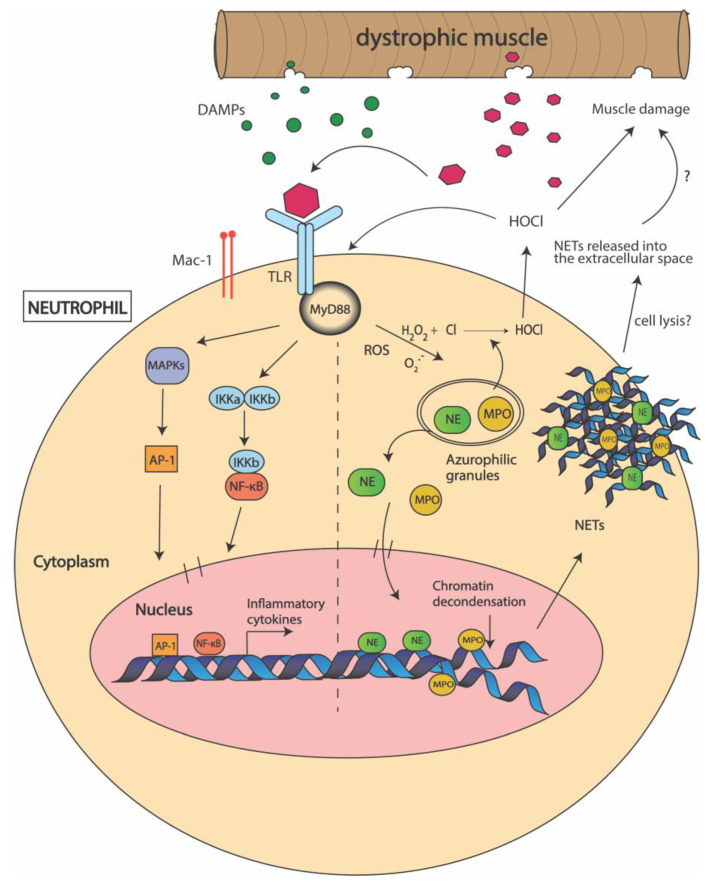
Mechanisms used by neutrophils to promote muscle damage in Duchenne muscular dystrophy (DMD). Following muscle damage, damage associated molecular patterns (DAMPS) are released from the dystrophic muscle and activate neutrophils via recognition by toll-like receptors (TLRs) and macrophage-1 antigen (Mac-1) on the cell surface. This interaction activates the myeloid differentiation primary response 88 (MyD88) pathway which further activates the IκB kinases (IKKs) and mitogen-activated kinases (MAPKs). This induces the expression of nuclear factor kappa B (NF-κB) and activator protein 1 (AP-1) transcription factors which promote the transcription of pro-inflammatory cytokines. DAMP-TLR interactions also cause the release of neutrophil elastase (NE) and myeloperoxidase (MPO) from the azurophilic granules within the neutrophil into the cytoplasm. MPO catalyzes the production of reactive oxygen species (ROS) including hypochlorous acid (HOCl), which elevates oxidative stress and promotes muscle cell lysis. NE induces chromatin decondensation and, together with MPO, lead to neutrophil extracellular trap (NET) formation. It is thought that NETs are released outside the cell by cell-lysis and further promote inflammation.

Neutrophils are essential players in regulating the process of tissue repair by aiding in the recruitment of macrophage subtypes which have a direct role in tissue regeneration [39]. Mature neutrophils contain different granules as well as several secretory vesicles that are filled with antimicrobial and tissue-destructive factors, making them equipped to assist in the defense response. The various mechanisms of defense include phagocytosis of damaged tissues, degranulation to release an arsenal of antimicrobial enzymes including neutrophil elastase (NE) and myeloperoxidase (MPO), and the most recently described DNA webs or neutrophil extracellular traps (NETs) [39,41,42] (Figure 2).

## 4. Does Myeloperoxidase (MPO) Production Contribute to DMD Pathogenesis?

In DMD muscle, neutrophils are activated within minutes after muscle damage [3,5]. Studies in *mdx* mice have shown that neutrophils recruited to the damaged site, release highly oxidative free radicals which lead to increased inflammation and oxidative stress [43]. One of these products is MPO, an enzyme produced predominantly by neutrophils and monocytes, which serves as a key component for antimicrobial defense assisting in phagocytosis [44]. MPO catalyzes the production of a potent oxidant, hypochlorous acid (HOCl) in the presence of hydrogen peroxide (H_2_O_2_) and chloride, which can increase oxidative stress. Oxidative radicals such as HOCl, can oxidize the thiol and carbonyl residues of vital cellular proteins of the sarcomere leading to the modification or loss of protein function, indicating that oxidative stress likely contributes to the pathophysiology of DMD [3,5,43] (Figure 2). MPO levels are significantly higher in *mdx* muscles and dystrophin-deficient dog (GRMD) muscles when compared to healthy muscles, suggesting that neutrophil-induced MPO might significantly contribute to muscle damage [43].

Therapies for DMD involving the depletion of neutrophils, or reducing oxidative stress via the reduction of MPO, have been recently investigated [45]. Taurine is a naturally occurring, cystine derived, amino acid having anti-inflammatory and antioxidant properties that are considered important for skeletal muscle function [43]. Feeding taurine to juvenile (14 days) *mdx* mice produced a significant reduction in the levels of MPO as compared to untreated *mdx* mice [46]. The decrease in the levels of MPO was associated with reduced muscle inflammation and necrosis providing further evidence that neutrophils are associated with the high inflammatory response and myonecrosis in DMD [46].

In addition to promoting oxidative stress, MPO is known to associate with the membranes of neutrophils via the macrophage-1 antigen (Mac-1) or CD11b/CD18 integrins. Activation of neutrophils by MPO induces the NF-κB and p38 MAPK signaling pathways [47]. Studies have shown that surface expression of CD11b was elevated in vitro after treatment with MPO, which promoted neutrophil degranulation and MPO release followed by superoxide production [47]. CD11b is a pan-immune cell receptor expressed on macrophages and neutrophils and regulates adhesion, migration, and induction of inflammatory responses [48,49]. CD11b expressing immune cells were reported in high numbers and suggested to promote inflammation in *mdx* mice [48,50]. However, the potential for integrin signaling to attenuate muscle damage by reducing inflammation in DMD is yet to be explored.

## 5. Can Neutrophil Elastase (NE) Be Used as a Target to Improve Muscle Regeneration in DMD?

NE is a serine protease mainly involved in the protection against pathogens [51]. However, NE can also cause detrimental effects, including extracellular matrix destruction, tissue fibrosis and mucus production [52]. Neutrophil accumulation and elevated levels of NE are characteristic features of acute lung injury, which is associated with increased inflammation and oxidative stress [53,54]. Treatment with NE inhibitors significantly reduced neutrophil accumulation and oxidative stress in vivo, suggesting a role for NE in promoting tissue damage via increased oxidative stress [54].

Proteomic analysis of *mdx* muscles identified that NE levels are elevated as compared to unaffected muscles [55]. It was suggested that NE impairs myoblast survival and proliferation in DMD by promoting degradation of cell adhesion molecules [55]. Therefore, it is possible that elevated levels of NE contribute to increased oxidative stress observed in DMD, however this has not been definitively shown (Figure 2). Consequently, therapies based on NE inhibition are currently under investigation.

## 6. Are Neutrophil Extracellular Traps (NETs) Formed in Dystrophic Muscle?

In addition to phagocytosis and degranulation, neutrophils are proficient in producing neutrophil extracellular traps (NETs). NETs are a complex of DNA and neutrophil-derived antimicrobial molecules including MPO and NE which help capture invading pathogens and cellular debris [56]. Although NETs are important for host defenses, they are known to promote tissue damage and necrosis in trauma, ischemic heart disease and various autoimmune disorders [57]. NET formation, termed “NETosis”, has been associated with inflammatory conditions involving neutrophil infiltration and recruitment, and is dependent on MPO, NE and peptidylarginine deaminase 4 (PAD4) and gasdermin D [56,58]. In fact, NETs are known to induce muscle fibrosis in ischemic reperfusion induced skeletal muscle injury by triggering the synthesis of cytokines, including IL-10 and TNF-α [59] (Figure 2). However, the association of NETs with muscle damage in DMD has not yet been characterized.

## 7. Impact of Aged Neutrophil Populations on Chronic Inflammation

Neutrophils are generally considered short-lived and only survive for a few hours in the circulation, after which they are cleared by a number of different processes. During normal muscle regeneration, neutrophils become apoptotic and can be phagocytosed by macrophages which abrogate further neutrophil recruitment and promote muscle repair [60]. Neutrophils can also leave the site of tissue damage and travel back to the bone marrow or lymph organs, in a process known as reverse migration, which ultimately results in the promotion of repair and resolution of inflammation [60]. Additionally, recent studies have demonstrated that neutrophils can re-enter the vasculature and are redistributed to other locations in the body, referred to as reverse transendothelial migration (rTEM) [61].

Several studies have reported that human neutrophils can survive for more than five days in the circulation, during which time they undergo extensive phenotypic changes [62,63]. Aged neutrophils show signs of a proinflammatory phenotype, including the upregulation of C-X-C chemokine receptor type 4 (CXCR4), promoting their clearance from the circulation [63]. However, in dystrophic muscle, failure of this process likely contributes to chronic inflammation. 

Aged neutrophils differ from those that are freshly released from the bone marrow with respect to their cellular receptors and morphology [39]. Specifically, aged neutrophils are characterized by increased expression of chemokine receptors, typically CXCR4, TLR4, intercellular adhesion molecule-1 (ICAM-1), Mac-1 (CD11b/CD18), and very-late antigen 4 or VLA4/CD49d integrins, which promote the adhesion of neutrophils to activated endothelial cells at the inflammation site [38,39]. These are prone to unregulated and frequent degranulation and increased ROS production as shown in many metabolic disorders and infections [39]. In fact, ITGβ2, a Mac-1β subunit, is differentially expressed in early stages in DMD patients [64]. In addition, ECM receptors including VLA-4 are upregulated in *mdx* mice, and Mac-1 and lymphocyte function-associated antigen-1 (LFA-1) levels are increased on circulating *mdx* neutrophils [65]. Thus, the increase in VLA-4 and Mac-1 expression associated with high neutrophil recruitment may suggest that there exists a larger proportion of aged neutrophils at the damaged sites, which might be responsible for the neutrophil-mediated muscle damage in DMD. 

## 8. Additional Factors Affecting Neutrophil Activation

An additional mechanism of persistent neutrophil activation is possibly driven by the fibrin and fibrinogen deposition which is a characteristic feature of the dystrophic muscle microenvironment after necrosis occurs [66]. Fibrinogen is a soluble acute phase protein which is released at the site of inflammation and helps to increase vascular permeability [67]. However, fibrinogen deposition in dystrophic muscle promotes neutrophil and macrophage recruitment through interactions with the Mac-1 integrin receptor. This interaction activates the NF-κB and c-Jun N-terminal kinase contributing to the manifestation of inflammation by upregulating the production of pro-inflammatory cytokines including IL-1β [66,68]. In addition, the interaction of fibrinogen with Mac-1 expressing neutrophils prolongs neutrophil survival by activating anti-apoptotic signaling pathways [69]. Thus, upregulated fibrin deposition may promote inflammation by constant recruitment of neutrophils in dystrophic muscle.

## 9. Conclusions

The persistent inflammatory state observed in DMD due to the continuous cycles of damage and repair of dystrophic muscle presents a unique environment for diverse neutrophil subpopulations to exist. Their production, maturation, release, and elimination are tightly regulated to maintain homeostatic stability and proper balance between antimicrobial and proinflammatory functions. Therefore, understanding the factors responsible for skewing neutrophil function towards the more “pathogenic” subtype is of great therapeutic interest. Further research into the critical roles of neutrophils throughout the inflammatory process in DMD will expand the possibilities of targeting neutrophils to reduce muscle weakness without compromising host defenses.
